# The Effect of the *Crocus Sativus* L. Carotenoid, Crocin, on the Polymerization of Microtubules, *in Vitro*

**Published:** 2013-01

**Authors:** Hossein Zarei Jaliani, Gholam Hossein Riazi, Seyyed Mahmoud Ghaffari, Oveis Karima, Abbas Rahmani

**Affiliations:** 1 Department of Biochemistry, Institute of Biochemistry and Biophysics (IBB), Tehran University, Tehran, Iran; 2 School of Biology, University College of Sciences, University of Tehran, Tehran, Iran

**Keywords:** Carotenoid, Fluorescence spectroscopy, Microtubules, Tubulin

## Abstract

***Objective(s): ***Crocin, as the main carotenoid of saffron, has shown anti-tumor activity both *in vitro* and *in vivo*. Crocin might interact with cellular proteins and modulate their functions, but the exact target of this carotenoid and the other compounds of the saffron have not been discovered yet. Microtubular proteins, as one of the most important proteins inside the cells, have several functions in nearly all kinds of cellular processes. The aim of this study was to investigate whether crocin affects microtubule polymerization and tubulin structure.

***Materials and Methods: ***Microtubules were extracted from sheep brains after two cycles of temperature-dependant assembly-disassembly in the polymerization buffer (PMG). Then phosphocellulose P11 column was used to prepare MAP-free tubulin. Turbidimetric assay of microtubules was performed by incubation of tubulins at 37 ºC in PIPES buffer. To investigate the intrinsic fluorescence spectra of tubulins, the emission spectra of tryptophans was monitored. To test the interaction of crocin with tubulin in more details, ANS has been used.

***Results: ***Crocin extremely affected the tubulin polymerization and structure. Ultraviolet spectroscopy indicated that crocin increased polymerization of microtubules by nearly a factor of two. Fluorescence spectroscopic data also pointed to significant conformational changes of tubulin.

***Conclusion: ***We showed that crocin increased tubulin polymerization and microtubule nucleation rate and this effect was concentration dependant. After entering cell, crocin can modulate cellular proteins and their functions. Concerning the results of this study, crocin would be able to affect several cell processes through interaction with tubulin proteins or microtubules.

## Introduction

Saffron (*Crocus sativus *L.) is one of the spices used for treatment of some diseases in traditional medicine. Most important components of saffron are carotenoids, crocetin, di-methyl crocetin and crocin. It is worthy to mention that crocin is di-gentiobioside ester of the crocetin. It has been reported that saffron extracts possess some health beneficial effects such as anti-oxidant, anti-tumor and cancer prevention. There are a lot of evidences which demonstrate that crocetin and its derivatives can inhibit cell growth and proliferation *in vitro *(1, 2). It has been proven that the growth of malignant human tumor cell lines K562 and HL60 can be inhibited by saffron carotenoids crocin, crocetin and also di-methyl crocetin (3). In other studies on breast cancer cell lines, different concentrations of saffron extracts have had a clear dose-dependent inhibitory effect on the cell proliferations. Moreover, this result was reported to be independent of estrogen receptor. Although there are additional reports about the anti-tumor effects of saffron carotenoids, the exact mechanism by which saffron extracts exert their anti-tumor effects has to be illustrated in more details (4). *In vitro *and *in vivo* studies demonstrated that anti-proliferative effects of crocin on T 24 cell line (human bladder carcinoma) was parallel with apoptotic effects which are also accompanied by morphologically detectable changes in the cells. A large number of crocin treated cells were abolished in G0/G1 transition of the cell cycle. Moreover, anti-apoptotic factors such as Bcl-2 and survivin were down-regulated in these cells (5). Anti-tumor activities of saffron extracts were well studied in tumor transplantated animal models. In these studies, administration of saffron extracts increased the lifespan of tumor bearing animals up to about two times (2). These carotenoids could inhibit pancreatic tumorigenesis in an athymic nude mice model. Apoptosis is also stimulated in such athymic nude mice (6). Although, many anti-tumor drugs has restrictions in use due to their genotoxicitys, saffron extract has not been reported to have such effects, and even it has succeeded in preventing genotoxic effects of some common drugs such as cisplatin, cyclophosphamide and mitomycin C (7). Another problem, in treatment of different types of tumors, is the toxic effects of the drug itself on the normal cells and tissues of the body. It has been shown that the saffron carotenoids do not have any toxic effect against normal and non-malignant cells; Despite that there are obvious anti-proliferative effects in the same concentrations of the carotenoids (8). Other studies demonstrated that saffron extracts and their compounds inhibited DNA and protein synthesis and there is evidence that the normal cells are much less affected by these carotenoids than cancer cell lines (9).

It seems that crocin is the most potent carotenoid of saffron extracts which has anti-tumor and cancer preventing effects (10). It has recently been shown that ethanolic extracts of saffron and specially its carotenoid crocin prevents the formation of papillomas in the mouse model of skin carcinogenesis. Besides, there is sufficient evidence that this prevention is not produced by gentiobiose or glucose alone (11).

Microtubules are one of the major components of cytoskeleton in nearly all eukaryotic cells. They contribute in many processes in cell namely mitosis, signal transduction and secretion of hormones (12, 13). By knowing the important role of the microtubules in the spindle formation during the mitosis, they have formed one of the most important targets of cancer therapy. There are many types of anti-tubulin agents which have anti-tumor properties. Several compounds from different sources can be found elsewhere (14), in addition to the well-known paclitaxel. The discovery of new microtubule binding agents and using them as anti-cancer agents, especially from the natural sources, has been continued until the last years, and reviewed by Yue *et al* (15). 

To explore the anti-tumor properties of crocin *in vitro*, we tested the effects of this carotenoid on the polymerization of microtubules. Microtubular proteins are extracted and purified from sheep brain tissues, and were polymerized in the presence and in the absence of crocin. After that, the structural changes of tubulins due to the interaction with crocin were analysed by fluorescence spectroscopy. 

## Materials and Methods

***Materials***

Crocin, purified from the *Crocus sativus *L. extract, was provided by Dr S Z Bathaei (Tarbiat Modares University, Tehran, Iran). EGTA, guanosine-5´-triphosphate (GTP), ATP, glycerol and MgSO_4_ were obtained from Sigma (Dorset, England). Phosphocellulose P11 was prepared from Whatman (Florham Park, USA). All other chemicals such as KOH, NaCl and ANS (Merck) were of analytical grade and used without further purification. Solutions were prepared with double distilled water and were kept at 4 ºC before use.

***Tubulin purification***

Microtubular proteins were prepared from sheep brains, and in the procedure PEM (100 mM PIPES, 1 mM EGTA, 2 mM MgSO_4_, pH 6.9) was used as homogenization buffer. Microtubules were extracted after homogenization by two cycles of temperature-dependant assembly-disassembly, by using PMG (100 mM PIPES, 2 mM MgSO_4_, 1 mM EGTA, 3.4 mM Glycerol and pH 6.9) as the polymerization buffer. Then phosphocellulose P11 column was used to prepare MAP-free tubulin, as the method of Weingarten *et al *by some modification (16). The fractions eluted from the column stored at -70 ºC for further investigation. The Bradford reagent (Bio-Rad, Hercules, USA) was used to determine protein concentration in each step and bovine serum albumin was the standard in this determination. 

***UV spectroscopy***

Turbidimetric assay of MT was carried out by incubation in cuvettes at 37ºC and in PIPES buffer in a thermostatically controlled UV spectrophotometer (Varian, Melbourne, Australia). The changes were measured at 350 nm wavelength. To assay the effect of crocin on the polymerization of microtubules, tubulins were pre-incubated with crocin at 4ºC for 10 min. The same experiment was performed to compare the effect of the crocin with paclitaxel. All the turbidimetric measurements were initiated by the addition of 1 mM GTP and warming the reaction up to 37ºC. 

***Fluorescence spectroscopy***

Intrinsic fluorescence spectra of MAP-free purified tubulins in the presence of crocin were measured to investigate the structural effects of this ligand on the tubulins. The emission spectra of tryptophans after excitation at 295 nm have been monitored at 1 nm precision. To test the interaction of crocin with tubulin in more details and to examine whether there is hydrophobic area exposed, 8-anilino-1-naphtalenesulfunic acid (ANS) has been used. Excitation wavelength was 380 nm and the emission spectra was monitored between 450-550 nm. All the measurements were performed at 25ºC and by 2 µM concentration of tubulin, in the absence or presence of the increasing concentrations of crocin. 

***Statistics***

The Student t-test was used to compare the means obtained from polymerized tubulins isolated from the four independent experiments.* P-*values< 0.05 were considered to be statistically signiﬁcant.

## Results

***Crocin effect on the MT assembly***

**Figure 1:**
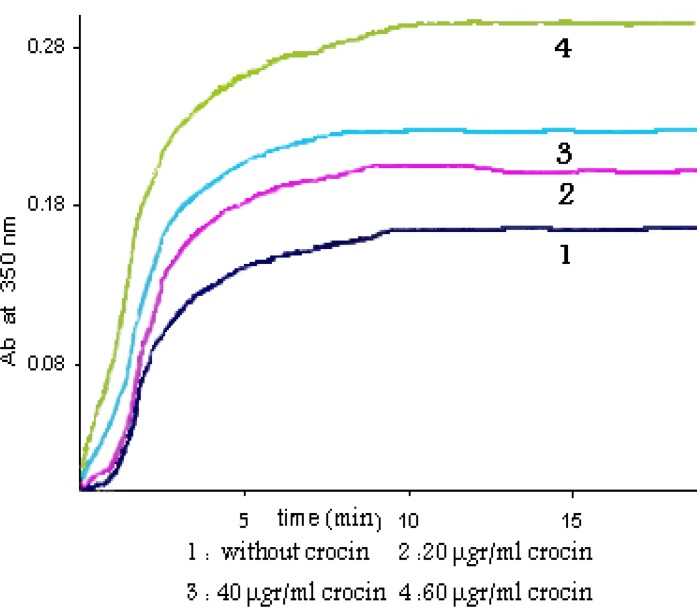
Crocin effect on the MT assembly MT proteins (2 µgr/ml) were pre-incubated at 4 ºC in PIPES buffer with increasing concentrations of crocin. Polymerization was initiated by adding 1 mM GTP. The turbidity was monitored at 350 nm at 37 ºC. 1, 0 µgr/ml crocin; 2, 20 µgr/ml crocin; 3, 40 µgr/ml crocin; 4, 60 µgr/ml crocin

The effect of crocin on the polymerization of microtubular proteins was measured as shown in Figure 1. In the presence of different concentrations of crocin there is an obvious increase in the MT assembly. The extent of MT assembly was increased by crocin, by nearly a factor of two (1.92). The difference between the means of polymerized microtubule levels in the crocin-treated and untreated tubulin groups was statistically significance (*P-*value< 0.001). 

**Figure 2:**
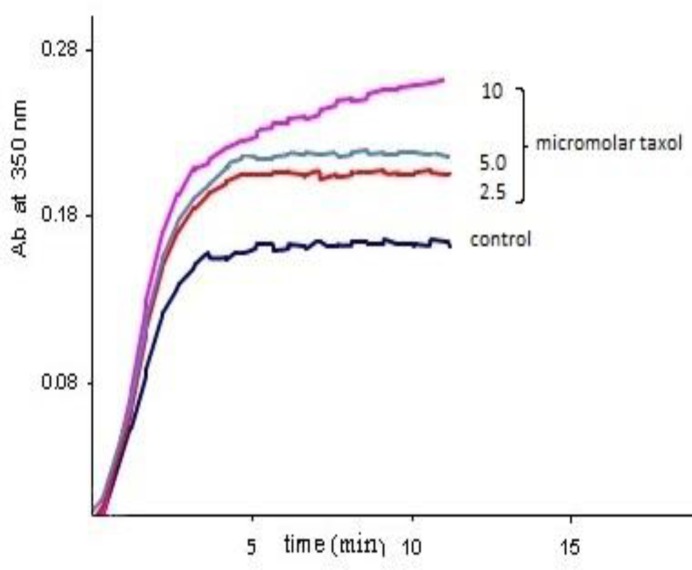
Paclitaxel effect on the MT assembly To compare the effect of crocin with a well-known ligand of tubulin, paclitaxel, microtubular proteins (2 µgr/ml) were again pre-incubated at 4ºC in PIPES buffer. Polymerization was initiated by adding 1 mM GTP. The turbidity was monitored at 350 nm at 37ºC. The increasing concentrations of paclitaxel up to 10 micromolar is demonstrated in the figure

 Also the time needed for the microtubules to nucleate was largely decreased. This result is comparable with the effect of paclitaxel which is demonstrated in Figure 2.

***MT repolymerization assay***

**Figure 3:**
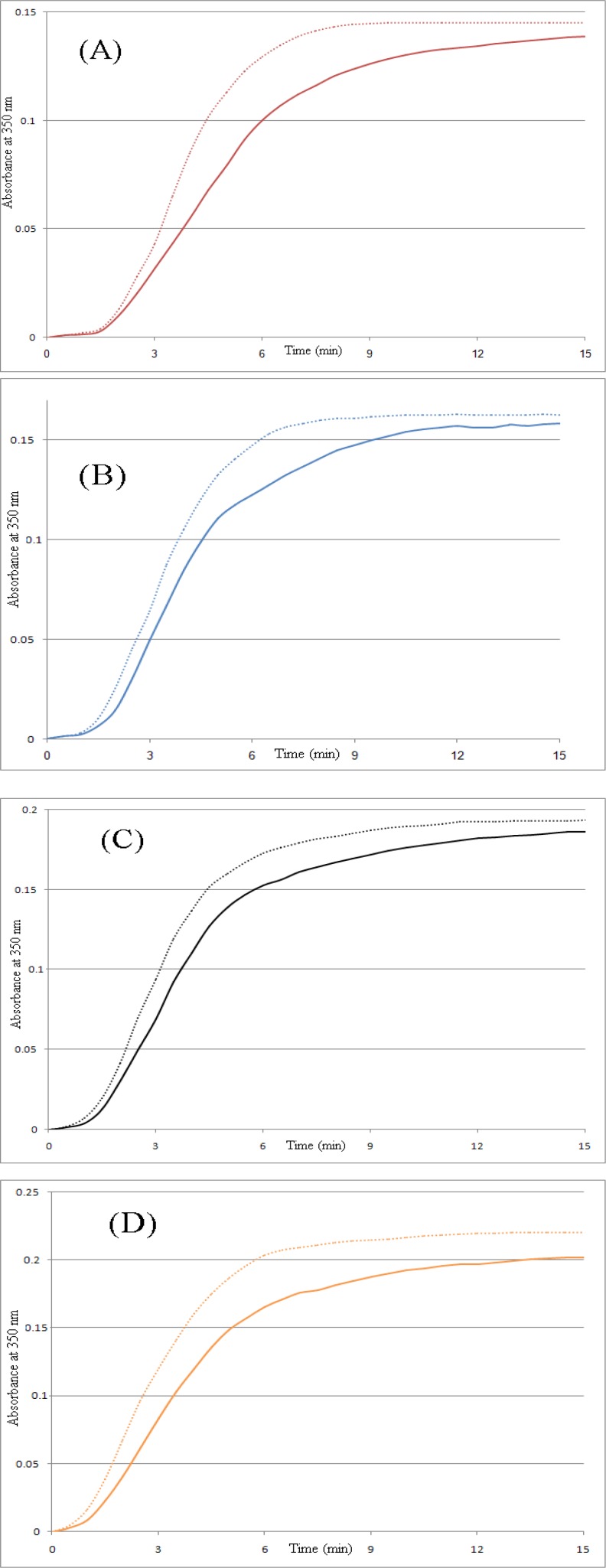
Tubulin repolymerization assay assembled microtubule proteins were incubated at 4 ºC for 30 min. Repolymerization of the microtubules was started by raising the temperature from 4 ºC to 37 ºC, without any further addition of guanosine-5'-triphosphate or crocin. Then turbidimetry was monitored by the change in absorbance at 350 nm (dashed lines). All solid lines are first polymerization assay curves. (A) 0 µg/ml crocin, (B) 15 µg/ml crocin, (C) 30 µg/ml crocin, (D) 45 µg/ml crocin

Assembled MTs were disassembled by cooling at 4ºC; re-warming the solution to 37ºC induced re-assembly of the microtubules, as in the control group. Repolymerization assay showed that depolymerized microtubules made polymers again after 30 min incubation in 4ºC (Figure 3). 

***Intrinsic fluorescence spectra***

**Figure 4:**
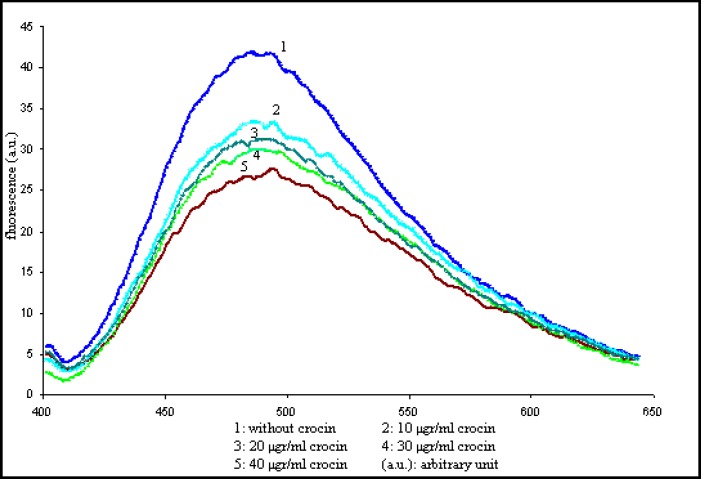
Intrinsic fluorescence spectra of 2 µM tubulin in the presence of increasing concentrations of crocin. The solution conditions were 1 M PIPES buffer, pH 6.9, containing 1 mM EGTA and 2 mM MgSO_4_. The excitation was at 295 nm and the emission spectra were monitored between 300 and 450 nm. 1, without crocin; 2, 10 µg/ml crocin; 3, 20 µg/ml crocin; 4, 30 µg/ml crocin; 5, 40 µg/ml crocin; 6, 50 µg/ml crocin; 7, 60 µg/ml crocin

To investigate the structural effects of crocin on the tubulin, the intrinsic fluorescence spectra of the protein was measured in the presence of crocin. As the result of crocin interaction with the tubulin, the fluorescence spectra of surface exposed tryptophans were quenched, and also there was a shift in the maximum wavelength of the emitted light, as shown in Figure 4.

***Decrease of ANS fluorescence by crocin-tubulin interaction***

**Figure. 5:**
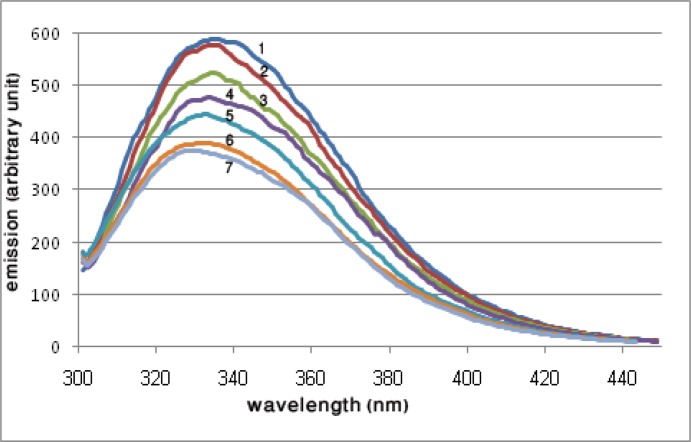
Emission spectra of tubulin-ANS complex by increasing concentrations of crocin Tubulin was mixed with different concentrations of crocin for 10 min at 4 ºC. Then ANS was added and treated for additional 5 min, and emission spectra of the complex was monitored between 400 and 650 nm

There are several low affinity sites and one high affinity site for the apolar molecule ANS on tubulin molecules. The strong fluorescence of tubulin-ANS complex is extremely environmentally sensitive. Therefore it is used to probe the conformational changes induced by the ligand. To investigate the nature of the crocin-tubulin interaction 2 µM tubulin was incubated in the presence of increasing concentrations of crocin for 10 min at 4ºC. ANS (50 µM final concentration) was added to tubulin-crocin and incubated for 5 min again. The concentration-dependant decrease in tubulin-ANS fluorescence was induces by crocin, as shown in Figure 5. Incubation of tubulin with ANS before the addition of crocin generated similar effects. 

## Discussion

In this study the effect of crocin, the main saffron carotenoid, was explored in an *in vitro *assay. At first the concentration of 0-60 µg/ml crocin was used. We showed that crocin increased tubulin polymerization and microtubule nucleation rate and this effect was concentration dependant (Figure 1). The procedure was described previously (17), and was then frequently used by some modifications. The absorbance spectra of crocin in the concentrations used was not above the baseline and did not interfere with the assay, so the frequently used 350 nm wavelength was monitored as the turbidimetry assessment in tubulin polymerization assay. 

As it was shown in Figure 1, crocin in the concentration of 60 µg/ml increased the polymerized microtubules by nearly a factor of two (1.92 times increase, and the *P-*value< 0.001). This effect is approximately equal to the level of seen by 10 µM of paclitaxel which is demonstrated in Figure 2. The same effect of the paclitaxel on polymerization of microtubules is reported frequently in the literature (18, 19). Curves of the Figures 1 and 2 are typical of experiments done at least four times. 

Assembled MTs were disassembled by cooling to 4 ºC and then reassembled by re-warming up to 37 ºC; MT repolymerization was observed (Figure 3). These results indicate that crocin did not affect MT repolymerization. Although some agents induce irregular aggregates of microtubules and cooling does not appear to depolymerize these aggregates. But crocin did not induce aggregate formation even at high concentrations. Microtubules were repolymerized after cooling to 4 ºC and heating to 37 ºC, and this means that the polymers were not assembled by denatured tubulin dimers and aggregates. The same results were produced by the other ligands such as titanium dioxide nanoparticles before (20). 

By using intrinsic fluorescence spectroscopy we explored changes induced in protein structure. Excitation at 295 nm was used and the changes in the emission wavelength showed alterations in the tryptophan environment. The results showed that crocin affected the polarity in the vicinity of the tryptophans, so that the fluorescence emission of the excited tryptophans had been quenched, and also a blue shift of about 10 nm was detected in the maximum wavelength of the spectra (Figure 4).

Fluorescence experiments with ANS demonstrated that crocin decreased fluorescence emission (Figure 5). Tubulin-ANS decrease in fluorescence may results from burring some of exposed tubulin's hydrophobic pockets for ANS binding. Alternatively crocin binding might induce a conformational change in tubulin leading to decreased tubulin-ANS fluorescence. Conformational changes in protein induced some hydrophobic pockets to hide inside the protein tertiary structure, decreasing tubulin-ANS fluorescence. GTP has fluorescence quenching ability (21), decreasing intrinsic fluorescence in the tryptophans emission spectra. GTP binding to tubulin has a crucial role in tubulin polymerization. Fluorescence data and Turbidimetric assays demonstrated that the conformational changes due to crocin binding to tubulin might affect GTP binding site conformation, so that the polymerization activity of tubulin was increased.

Tubulin is an important cytoplasmic protein with a variety of functions, from the neurotransmitter transport in the nerve cells to cell division and shape in other cell types. Many anti-tumor drugs have already been introduced which exert their functions through microtubules stabilization or disruption. Once crocin enters the cell, it can modulate cellular proteins and alter their functions. 

## Conclusions

Concerning the results of this study, crocin would be able to affect several cell processes through interaction with tubulin proteins or microtubules. 
